# Acceptance of using digital technologies to aid the timely detection of dementia: perspectives from Boston University Alzheimer’s Disease Research Centre

**DOI:** 10.1002/alz.089699

**Published:** 2025-01-09

**Authors:** Emily Beswick, Sarah Wilson, Zachary T Popp, Salman Rahman, Raiyan R Khan, Clare Tolley, Spencer Low, Sarah Slight

**Affiliations:** ^1^ Newcastle University, Newcastle Upon Tyne UK; ^2^ Boston University Chobanian & Avedisian School of Medicine, Boston, MA USA; ^3^ Stanford University School of Medicine, Stanford, CA USA

## Abstract

**Background:**

Using digital technologies to detect the early signs of dementia‐causing diseases could improve the timely detection of these diseases. To support this approach, we explored users’ perspectives on the acceptability of using a variety of digital technologies.

**Method:**

A sub‐group of participants from Boston University’s Alzheimer’s Disease Research Centre were recruited. Participants used up to nine study technologies (active smartphone apps (n=3), passive apps (n=2), wrist worn activity monitor (n=2), EEG headband (n=1), Oximeter ring (n=1)) for two weeks, every three‐months, over a year. Semi‐structured interviews were conducted after the first two weeks. A framework thematic analysis approach was applied to the interview transcripts, assisted by N‐Vivo (QSR, version 14.23.2). The Technology Acceptance Model^(1)^ aided in refining key themes.

**Result:**

Twenty‐four individuals were interviewed: 13 with mild cognitive impairment (MCI) and 11 without cognitive impairments. Three key themes emerged; perceived usefulness, intention to use and enjoyment of use. Participants were excited about the possibility of using digital technologies to remotely monitor their brain health, but some raised concerns that this could reduce personal interaction with their healthcare professional. Participants with pre‐existing health conditions, questioned the accuracy of the technology. For example, a participant wondered what effect an oxygen mask used for sleep apnoea would have on the data collected from the oximeter ring, reducing their perceived usefulness of the devices. Many participants using seven or more devices reflected on the burden of using multiple devices, finding it was challenging to familiarise themselves with them and then remember to use them all. Participants with MCI enjoyed the cognitive stimulation they felt after using the gamified cognitive tasks in the active smartphone apps, whereas participants without cognitive impairment wanted more variety to improve engagement.

**Conclusion:**

Results highlight the acceptability of a variety of digital technologies for the timely detection of dementia‐causing diseases and discussed the burden of using multiple devices. Future research should explore how pre‐existing health conditions could affect the accuracy of digital technologies to improve real world applicability.

References

Davis FD. Perceived usefulness, perceived ease of use, and user acceptance of information technology. MIS quarterly. 1989 Sep 1:319‐40.

